# PAI-1 is a potential transcriptional silencer that supports bladder cancer cell activity

**DOI:** 10.1038/s41598-022-16518-3

**Published:** 2022-07-16

**Authors:** Hideki Furuya, Yuka Sasaki, Runpu Chen, Rafael Peres, Kanani Hokutan, Kaoru Murakami, Nari Kim, Owen T. M. Chan, Ian Pagano, Lars Dyrskjøt, Jørgen B. Jensen, Per-Uno Malmstrom, Ulrika Segersten, Yijun Sun, Abolfazl Arab, Hani Goodarzi, Steve Goodison, Charles J. Rosser

**Affiliations:** 1 Department of Biomedical Science, Cedars-Sinai Medical Center, Samuel Oschin Comprehensive Cancer Institute, Los Angeles, CA 90048 USA; 2 Department of Microbiology and Immunology, State University of New York at Buffalo, Buffalo, NY 14260 USA; 3 Clinical and Translational Research Program, University of Hawaii Cancer Center, Honolulu, HI 96813 USA; 4 Department of Molecular Biosciences and Bioengineering, University of Hawaii at Manoa, Honolulu, HI 96813 USA; 5 Cancer Prevention in Pacific Program, University of Hawaii Cancer Center, Honolulu, HI 96813 USA; 6 Department of Molecular Medicine, Aarhus University Hospital, 8200 Aarhus, Denmark; 7 Department of Clinical Medicine, Aarhus University, 8200 Århus, Denmark; 8 Department of Urology, Aarhus University Hospital, 8200 Århus, Denmark; 9 Department of Surgical Sciences, Uppsala University, 751 05 Uppsala, Sweden; 10 Department of Biochemistry and Biophysics, Department of Urology, University of California San Francisco, San Francisco, CA 94143 USA; 11 Quantitative Health Sciences, Mayo Clinic Florida, Jacksonville, FL 32224 USA

**Keywords:** Bladder cancer, Cancer genetics, Oncogenes

## Abstract

The extracellular activity of Plasminogen activator inhibitor-1 (PAI-1) is well described, acting as an inhibitor of tissue plasminogen activator and urokinase-type plasminogen activator, impacting fibrinolysis. Recent studies have revealed a pro-tumorigenic role of PAI-1 in human cancers, via the regulation of angiogenesis and tumor cell survival. In this study, immunohistochemical staining of 939 human bladder cancer specimens showed that PAI-1 expression levels correlated with tumor grade, tumor stage and overall survival. The typical subcellular localization of PAI-1 is cytoplasmic, but in approximately a quarter of the cases, PAI-1 was observed to be localized to both the tumor cell cytoplasm and the nucleus. To investigate the potential function of nuclear PAI-1 in tumor biology we applied chromatin immunoprecipitation (ChIP)-sequencing, gene expression profiling, and rapid immunoprecipitation mass spectrometry to a pair of bladder cancer cell lines. ChIP-sequencing revealed that PAI-1 can bind DNA at distal intergenic regions, suggesting a role as a transcriptional coregulator. The downregulation of PAI-1 in bladder cancer cell lines caused the upregulation of numerous genes, and the integration of ChIP-sequence and RNA-sequence data identified 57 candidate genes subject to PAI-1 regulation. Taken together, the data suggest that nuclear PAI-1 can influence gene expression programs and support malignancy.

## Introduction

Cancer of the urinary bladder can be aggressive and develop resistance to currently available cancer therapies. An estimated 83,730 newly diagnosed cases of bladder cancer (BCa) and 17,200 deaths from BCa occurred in 2021 in the US alone^1^. Plasminogen activator inhibitor type-1 (PAI-1) has been identified as one of a panel of 10 urine-based diagnostic biomarkers that forms the basis of a clinical test for the non-invasive detection of BCa^2–4^. The best described function of PAI-1 is as an inhibitor of tissue plasminogen activator (tPA) and urokinase-type plasminogen activator (uPA), factors involved in the conversion of plasminogen to active plasmin, which in turn mediates fibrin clot hydrolysis^5^. Thus, PAI-1 can play a major role in benign disorders such as deep vein thrombosis, myocardial infarction, atherosclerosis, and stroke^6^. PAI-1 expression is regulated by a number of intrinsic factors (e.g., transforming growth factor-β, interleukin 6 and tumor necrosis factor-α) and extrinsic factors (e.g., cellular stress)^7,8^.

In cancer research, uPA was originally highlighted because multiple clinical studies showed that cancer patients with a high expression of uPA, and its receptor uPAR, have worse clinical outcomes^9–11^. Since PAI-1 is an inhibitor of uPA, it was expected that PAI-1 would have an anti-tumor effect. However, accumulating evidence supports a pro-tumorigenic role of PAI-1 in cancer^8,12–16^. In previous work, we have shown that the reduction of PAI-1 expression in bladder cancer cell lines (T24 and UM-UC-14) by stable knockout, or via the PAI-1 small molecule inhibitor, tiplaxtinin, significantly reduced colony formation and cell proliferation, and induced apoptosis. Conversely, the overexpression of PAI-1 augments cellular proliferation *in vitro.* These results were confirmed in an animal model in which tumors formed by cells with no PAI-1 expression were smaller in size and had reduced rates of proliferation and angiogenesis compared to tumors with forced PAI-1 expression^17^. In a follow-up study, we showed that cell cycle arrest was associated with the downregulation of PAI-1. The arrest was accompanied by the depletion of G_1_ cell cycle transition complexes, cyclin D3/cyclin-dependent kinase (CDK) 4/6 and cyclin E/CDK2, and the up-regulation of the cell cycle inhibitors p27^kip1^, p21^Cip1/Waf1^ and p53^18^. These findings confirm the importance of PAI-1 in the regulation of tumor cell growth and survival.

Previous reports have established that PAI-1 protein is secreted and is localized in the extracellular space, including the extracellular matrix (ECM) and extracellular exosomes^19–21^. Described PAI-1 functions in cancer have been focused on the extracellular functions mediated through the inhibition of uPA action, the binding to low-density lipoprotein receptor-related protein (LRP1) to initiate intracellular signaling, and the binding to vitronectin to inhibit cell attachment^8,22,23^. Reports have also shown that PAI-1 can be internalized and is able to interact with caspases or other apoptotic proteins within the cytoplasm^24^.

In this study, we initially investigated the expression of PAI-1 in a large cohort of bladder cancer tissues. Immunohistochemical staining of 939 human bladder cancer specimens showed that PAI-1 expression levels correlated with tumor grade, tumor stage and overall patient survival. Although the typical cytoplasmic subcellular localization of PAI-1 was evident, we noted that PAI-1 was also localized to the nucleus in ~25% of cases, an observation not previously reported, prompting us to investigate a possible nuclear function of PAI-1. A combination of techniques including, chromatin immunoprecipitation (ChIP)-sequencing, RNA-sequencing and microarray-based gene expression profiling, plus rapid immunoprecipitation mass spectrometry, were employed to provide insights into the possible role of nuclear PAI-1 and its impact on cancer cell gene expression profiles.

## Results

### PAI-1 expression is associated with aggressive bladder cancer

**Figure 1:**
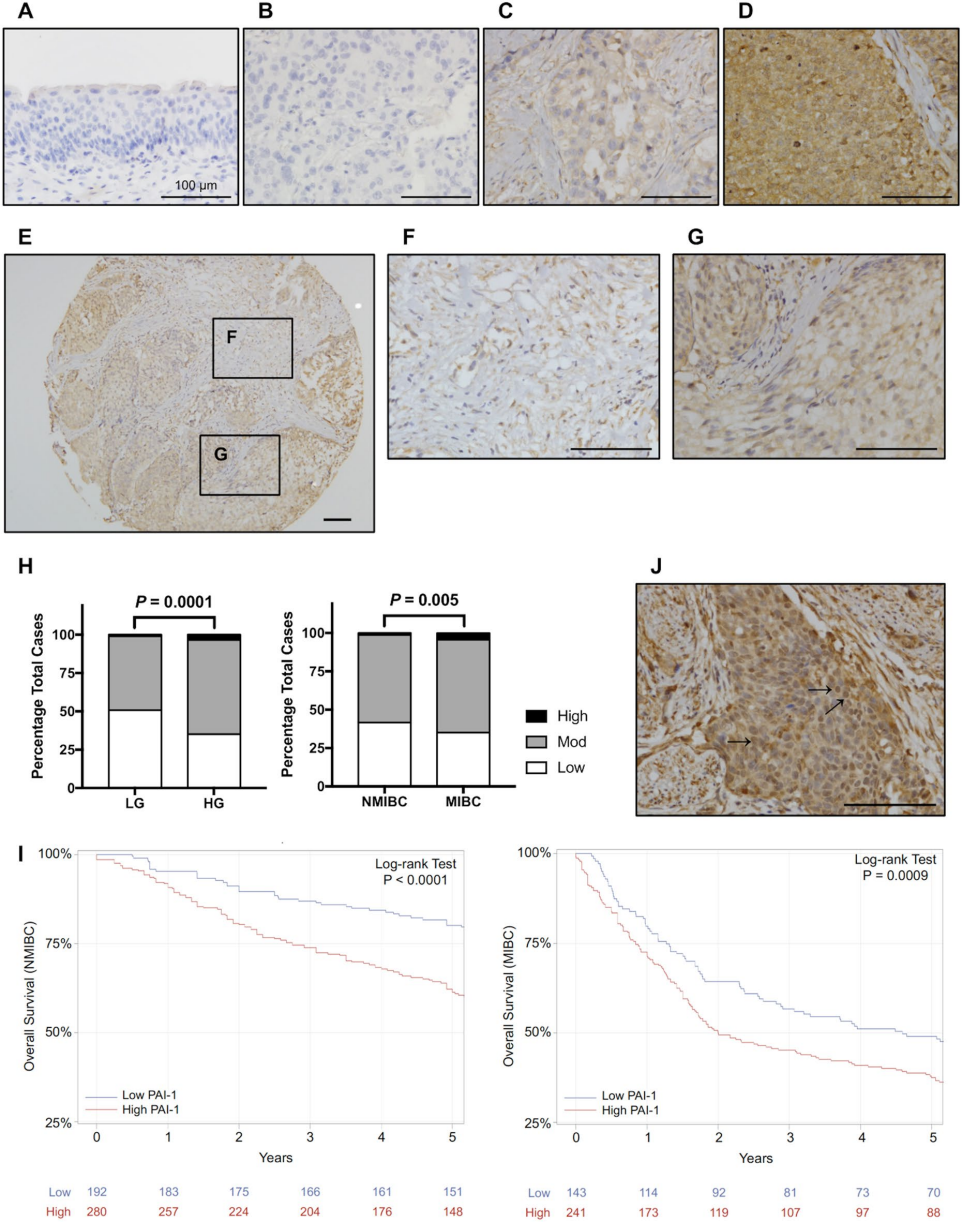
PAI-1 expression is increased in bladder cancer. Representative images of normal urothelium (**A**) and of bladder cancers with absent (**B**), weak (**C**) and strong (**D**) PAI-1 expression (brown) are shown. Representative images of a sample TMA core (**E**), PAI-1 expression in the tumor-associated stroma (**F**) and PAI-1 expression in the tumor epithelia (**G**). Scale bars, 100 μm. (**H**) Quantification of PAI-1 expression levels in low-grade (LG) *vs*. high-grade (HG) and NMIBC *vs*. MIBC. (**I**) Correlation between PAI-1 expression and overall survival in NMIBC and MIBC. Data are from analysis of a TMA containing 939 bladder tumors. (**J**) PAI-1 expression is noted in the nucleus and cytoplasm.

To investigate the clinical importance of PAI-1 expression in human bladder tumors, we performed immunohistochemical staining of a large excised tissue cohort (n = 939) including cohorts from Denmark (N = 587) and Sweden (N = 352). Detailed clinical information including demographic, clinical, disease and treatment characteristics are available in Table 1. The median follow-up for the entire cohort was 5.37 years. Representative tumor tissue staining is shown in Figure 1A–G. The analysis of a relationship between immunochemical features and tumor grade showed that a higher expression level of PAI-1 was associated with high-grade disease, compared to that of low-grade disease (N = 863, *P* = 0.0001; Fig. 1H left). Analysis of the relationship between immunochemical features and tumor stage revealed that the expression level of PAI-1 in muscle invasive bladder cancer (MIBC) was higher (N = 872, *P* = 0.005; Fig. 1H right) than that of non-muscle invasive bladder cancer (NMIBC). Importantly, a higher expression level of PAI-1 was associated with worse overall survival both in NMIBC and MIBC (*P *< 0.0001 and *P *< 0.001, respectively; Fig. 1I). Analysis of PAI-1 mRNA levels in the TCGA-BLCA dataset (n = 412 bladder tumors) corroborated the immunohistochemical results (Supplementary Figure S1). A higher expression level of PAI-1 mRNA was associated with worse overall survival (Supplementary Figure S1A) and progression-free survival (Supplementary Figure S1B). Interestingly, when reviewing the immunohistochemical data, we noted that PAI-1 was typically expressed in the cytoplasm as expected, but that it was also present in the nucleus of ~25% of the BCa specimens (Fig. 1J).

**Table 1:** Demographic, clinical, and pathologic characteristics of the 939 subjects comprising the study cohort.

Features	Denmark N = 587	Sweden N = 352	All patients N = 939
**Age (years)**			
≤ 65	274 (47%)	90 (26%)	364 (39%)
> 65	313 (53%)	256 (73%)	569 (61%)
Unavailable	0 ( 0%)	6 ( 2%)	6 ( 1%)
**Sex**			
Female	144 (25%)	85 (24%)	229 (24%)
Male	443 (75%)	264 (75%)	707 (75%)
Unavailable	0 ( 0%)	3 ( 1%)	3 ( 0%)
**Tumor grade**			
Low	139 (24%)	84 (24%)	223 (24%)
High	437 (74%)	265 (75%)	702 (75%)
Unavailable	11 ( 2%)	3 ( 1%)	14 ( 1%)
**Tumor stage**			
Ta or Tis	139 (24%)	119 (34%)	258 (27%)
T1	133 (23%)	117 (33%)	250 (27%)
T2	294 (50%)	89 (25%)	383 (41%)
T3 or T4	21 ( 4%)	23 ( 7%)	44 ( 5%)
Unavailable	0 ( 0%)	4 ( 1%)	4 ( 0%)
**Lymph nodes**			
N0 or Nx	470 (80%)	341 (97%)	811 (86%)
N1	117 (20%)	11 ( 3%)	128 (14%)
**Progression**			
No	318 (54%)	230 (65%)	548 (58%)
Yes	269 (46%)	110 (31%)	379 (40%)
Unavailable	0 ( 0%)	12 ( 3%)	12 ( 1%)
**Follow-up (years)**			
Median	6.83	3.75	5.37

### Nuclear localization of PAI-1 and binding to chromosomal DNA

**Figure 2:**
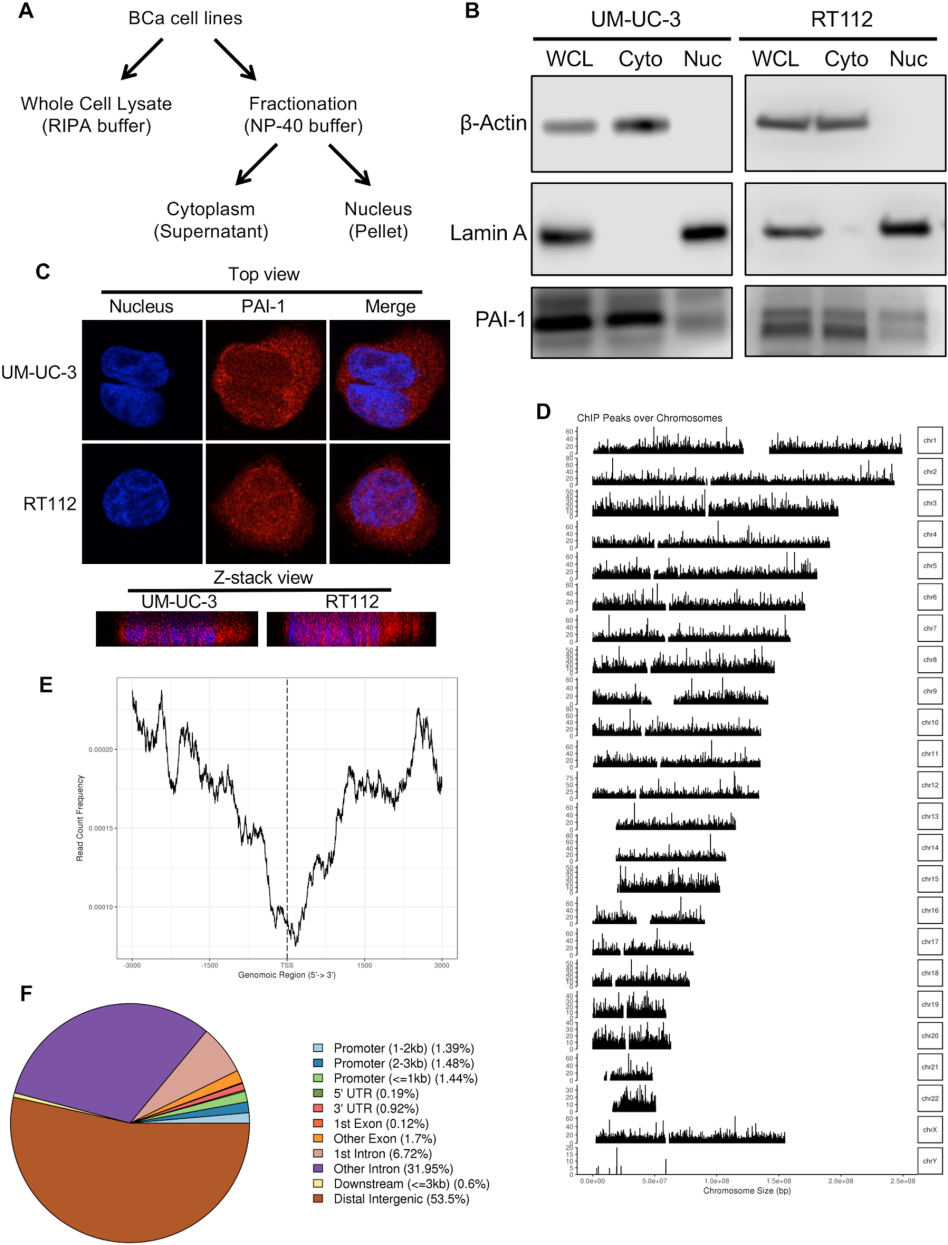
PAI-1 subcellular localization and ChIP-seq profiling. (**A**) Depicted is the cell fractionation protocol for isolating cytoplasm and nuclear protein. (**B**) All fractions including whole cell lysate (WCL), nuclear (Nuc), or cytoplasmic extracts (Cyto) from UM-UC-3 and RT112 cells were evaluated by immunoblot analysis. (**C**) Subcellular localization of PAI-1 (red) was visualized using specific antibody. (**D**) Histogram demonstrating distribution of PAI-1 binding across the genome in UM-UC-3 cells. The frequency of PAI-1 binding across chromosomes was calculated by dividing the number of probe sets per chromosome by the number of probe sets bound by PAI-1 with FDR < 0.2. (**E**) Histogram demonstrating relative PAI-1 bound peak location with respect to chromosome region in UM-UC-3 cells. (**F**) Pie chart of the genomic location distribution of PAI-1 in UM-UC-3 cells. This plot shows the percentage for each genomic location category. The categories are sorted by descending percentage.

To corroborate the nuclear presence of PAI-1, we examined PAI-1 cellular distribution in the nuclear and cytosolic fractions (Fig. 2A) of UM-UC-3 and RT112 cells by immunoblot analysis. Endogenous PAI-1 protein was readily detectable in both cellular components in both bladder cell lines (Fig. 2B). Subsequent immunofluorescence (IF) analysis confirmed the distribution of PAI-1 in both the cytoplasm and nucleus of UM-UC-3 and RT112 cells, although the signal appeared more prominent in the cytoplasm when observing whole cell distribution (Fig. 2C). Taken together, these results show that PAI-1 is present in the nucleus of these bladder cancer cells.

To determine if PAI-1 protein interacts with genomic DNA, we performed ChIP on nuclear extracts from UM-UC-3 and RT112 using an antibody specific to endogenous PAI-1, and sequenced the associated DNA (ChIP-seq, GSE201515). Endogenous PAI-1 binding was found on all chromosomes in both cell lines (Fig. 2D, Supplementary Figure S2 A). A count frequency analysis found that PAI-1 primarily binds to distal intergenic regions, but not gene promoters, suggesting that PAI-1 might function as a co-regulator rather than a classic transcription factor (Fig. 2E,F; Supplementary Figure S2 B & C). To evaluate the potential interaction of nuclear PAI-1 with other nuclear proteins, a RIME assay was conducted by Active Motif using material from both parental bladder cell lines. Filtered comparison in a Venn-diagram (http://bioinformatics.psb.ugent.be/webtools/Venn/) between anti-PAI-1 and IgG control antibodies identified 13 proteins that were pulled-down with nuclear PAI-1 (Supplementary Table S1). None of the identified co-precipitated proteins were known transcription factors or coregulators.

### Identification of potential nuclear PAI-1 regulated genes by expression profiling using RNA-seq

**Figure 3:**
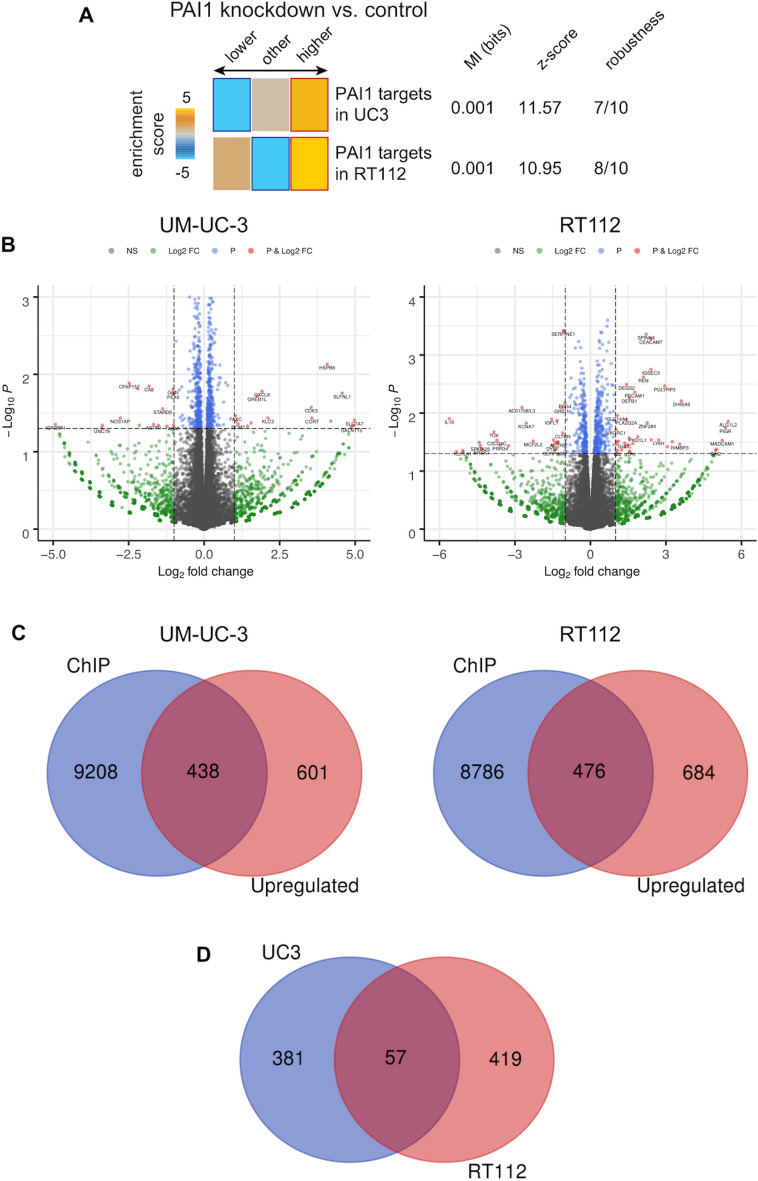
Gene expression profiling by RNA-seq and integration of ChIP-seq and RNA-seq. (**A**) Enrichment Analysis of RNA-seq indicate that knockdown of PAI-1 results in upregulation of the genes. (**B**) Volcano plot showing fold-change and p-value for the comparisons of negative control and PAI-1 siRNA. (**C**) Venn diagram showing the overlapping genes between ChIP-seq and upregulated genes from RNA-seq. (**D**) Venn diagram representing the overlap of nuclear PAI-1 regulating genes in UM-UC-3 and RT112 cells.

To test the downstream effects of PAI-1, we depleted PAI-1 by siRNA in UM-UC-3 and RT112 human bladder cell lines and subjected these cells to gene expression analysis using RNA-seq (GSE201517). PAI-1 downregulation by siRNA was performed in 3 independent replicate experiments and confirmed at both the mRNA and protein level. Enrichment analysis found that PAI-1 depletion resulted in an overall upregulation of gene expression (Fig. 3A), suggesting that PAI-1 may function in the suppression of gene expression.

A volcano plot analysis, using a fold-change cutoff of 2 and p-value threshold of 0.05 (Fig. 3B), identified differentially expressed genes (DEGs) in UM-UC-3 and RT112 cells. To identify genes that may be directly regulated by nuclear PAI-1, we identified genes that were identified as adjacent to PAI-1 binding by ChIP-seq, and overlapped the ChIP-seq and RNA-seq lists to produce a final common list containing 438 and 476 genes in UM-UC-3 and RT112, respectively (Fig. 3C; Supplementary Table S2 and S3). The overlap of the common lists from UM-UC-3 and RT112 identified 57 genes that are most likely to be regulated by nuclear PAI-1 (Fig. 3D; Supplementary Table S4). A second ChIP-on-chip experiment confirmed PAI-1 binding in the vicinity of the 57 genes, supporting the likelihood that these genes are regulated by nuclear PAI-1.

**Figure 4:**
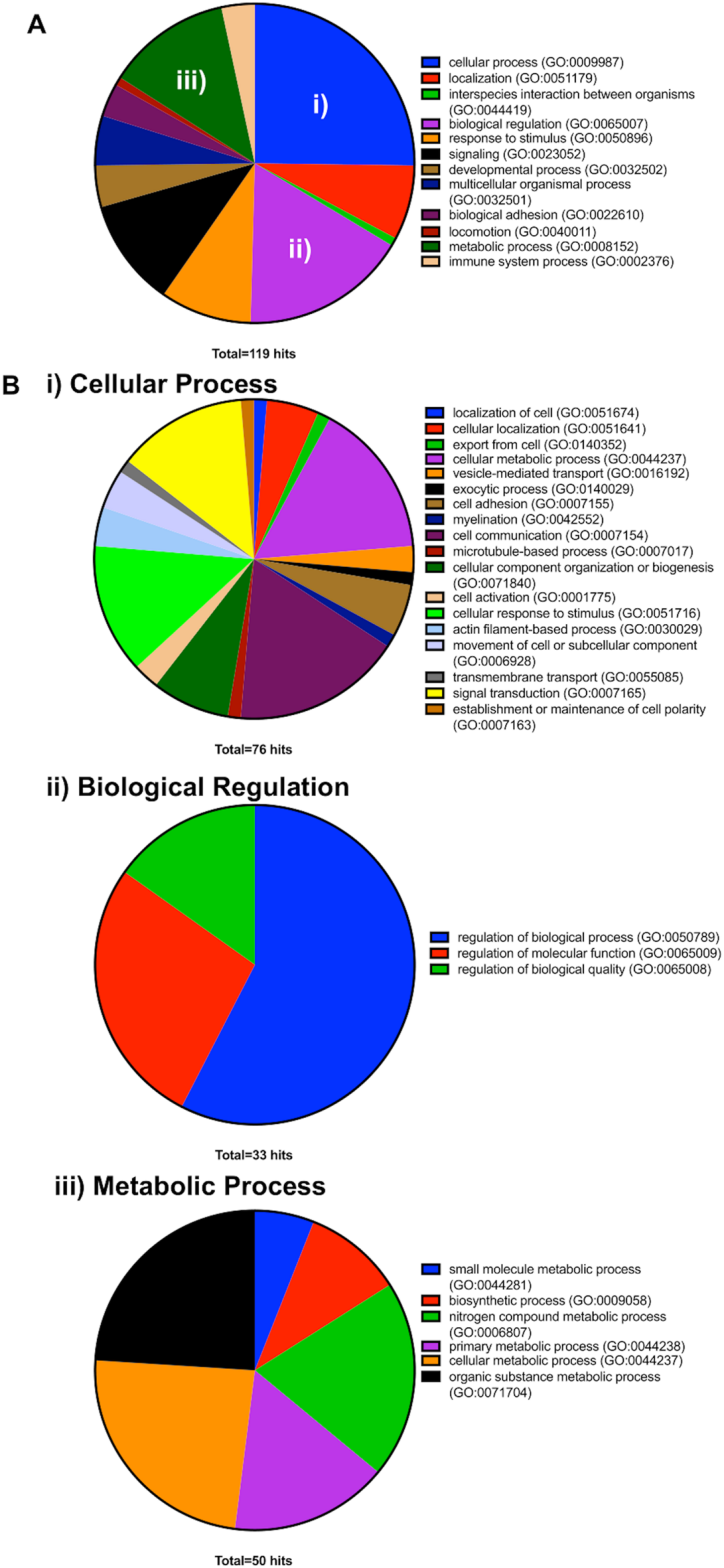
Gene ontology analysis of 57 nuclear PAI-1 regulating genes. (**A**) Biological process and (**B**) details of biological process including (i) cellular process, (ii) biological regulation, and (iii) metabolic process.

Gene ontology (GO) terms using Panther tools (http://www.pantherdb.org/tools/) revealed that 55 of the 57 genes are involved in biological processes, including (i) cellular processes, (ii) biological regulation, and (iii) metabolic processes (Fig. 4A). In the cellular process category, cell communication, cellular metabolic processes, cellular response to stimuli, and signal transduction were prominent (Fig. 4B-i). In the biological regulation group, the regulation of biological processes, molecular function, and biological quality were ranked in that order (Fig. 4B-ii). In the metabolic process category, cellular metabolic processes, organic substance metabolic processes, and primary metabolic processes were the most prominent (Fig. 4B-iii). The results suggest that genes regulated by PAI-1 are involved in diverse cellular activities.

## Discussion

In the initial part of our study, IHC analysis of 939 human BCa tumor tissue specimens revealed that PAI-1 expression levels correlated with tumor grade, tumor stage and overall survival. In the process of reviewing tissues, we also found that PAI-1 can be found localized to the nucleus as well as the cytoplasm. This prompted us to investigate a potential role for nuclear through the application of a panel of molecular profiling techniques. ChIP-seq analysis showed that nuclear PAI-1 binds to distal intergenic region DNA in both UM-UC-3 and RT112. Enrichment score analysis based on RNA-seq data showed that PAI-1 depletion resulted in the upregulation of numerous genes, indicating that PAI-1 may function as a silencer of gene expression programs. Integration of data from ChIP-seq and RNA-seq identified 57 genes as candidates for regulation by nuclear PAI-1. GO analysis shows that these genes are involved in multiple cellular processes. Shifts in the expression of specific gene subsets by nuclear PAI-1 may support key functions involved in cancer cell survival.

Previous studies have described that PAI-1 primarily localizes in the extracellular space, including the extracellular matrix (ECM) and extracellular exosomes^19–21^. This is confirmed by the publicly available database, The Human Protein Atlas (http://www.proteinatlas.org), however, another publicly available database, GeneCards (http://www.genecards.org), has stated that PAI-1 may also localize to nucleus, but without reference to any evidence. In our study, we employed two different approaches, IF and subcellular fractionation followed by western blot, to confirm the subcellular localization of PAI-1 to the nucleus and the cytoplasm in a subset of bladder cancer patients (Fig 1A–J). Confocal microscopic observations found positive staining in the nucleus and western blot analysis found PAI-1 in the nuclear fraction.

Accumulating evidence indicates that PAI-1 induces tumor cell growth and protects tumor cells from apoptosis^8,17,18,25,26^. The mechanism through which extracellular PAI-1 is involved in tumor cell growth and apoptosis is relatively well characterized, but a role for intracellular PAI-1, especially nuclear PAI-1, in cancer is unclear. ChIP-seq assays demonstrated the widespread binding of PAI-1 across chromosomal DNA (Fig 2D–F). Previously, Stefansson *et al.* suggested that PAI-1 may randomly bind to the DNA backbone^27^. ChIP-seq assays from this study showed that PAI-1 primarily binds to intronic and distal intergenic regions, with the majority of PAI-1 bound peaks being observed in proximal promoter regions (− 1000 bp).

Previous studies have demonstrated that PAI-1 has three specific protein-binding domains^28^, providing the ability to interact with multiple proteins^8^. In addition, DNA binding prediction analysis using DNAbinder^29^, DNABIND^30^ and Dr PIP^31^ suggested that PAI-1 is a non-DNA-binding protein. On the other hand, it has been reported that proteins (enhancers or silencers) binding to distal intergenic region can stimulate/suppress transcription initiation by communicating with promoters^32,33^. All these facts together led us to the hypothesis that nuclear PAI-1 regulates the transcription of genes as a distal enhancer/silencer. To test this hypothesis, we next performed RNA-seq followed by enrichment score analysis. The results suggest that PAI-1 functions as a silencer to inhibit these gene expression. Overlapping of the data from ChIP-seq and RNA-seq identified 57 genes that are potentially regulated by nuclear PAI-1 binding to distal intergenic region. Interestingly, GO analysis with the 57 genes revealed that these genes are involved in maintenance of normal cellular activity. Unlike normal cells, cancer cells grow uncontrollably by getting abnormal regulation of cell growth, cell cycle, apoptosis, and metabolism^34^. Thus, the results suggest that upregulation of nuclear PAI-1 suppresses the expression of these 57 genes, leading to the normal cellular activities. Additionally, PAI-1 tumoral expression was associated with a shorter overall survival in bladder cancer patients (Fig 1). Taken together, our data from this study represents the first landscape of PAI-1-chromosomal interactions in human bladder cancer, revealing a potential novel non-canonical function for PAI-1 in regulating gene expression.

This study has certain limitations. First, this is a retrospective study using valuable limited clinical specimen, thus the validation study using the same cohort cannot be performed. Second, there is no good model (*in vitro* nor *in vivo*) to replicate our findings because the function of nuclear PAI-1 may be complicated. Improving these aspects for future in-depth studies could further increase the persuasiveness of these results.

## Conclusion

It is known that proteins binding to distal intergenic regions can stimulate/suppress transcription initiation by communicating with spatially promoters^32,33^. Our analyses suggest that PAI-1 functions overall as a silencer of gene expression and we were able to identify a set of 57 genes that are potentially regulated by nuclear PAI-1. Additional work is required to identify whether the influence of nuclear PAI-1 is mediated through direct DNA binding or through interaction with secondary protein complexes, but the combination of data from IHC, ChIP and gene expression analysis, provide a foundation for the study of a role for nuclear PAI-1 in cancer cell behavior.

## Methods

### Patients and clinicopathologic information

The study was performed after approval by the Western Institutional Review Board (IRB # 20141019) under a request of waiver of informed consent on archived pathologic specimens. All methods were carried out in accordance with relevant guidelines and regulations. Tissue microarrays (TMAs) associated with two large patient cohorts were used in the current study. Cohort 1 was comprised of 587 bladder tumors from Aarhus University Hospital (Aarhus, Denmark, IRB # 1706291, National Committee on Health Research Ethics)^35^ and cohort 2 was comprised of 352 bladder tumors from Uppsala University Hospital (Uppsala, Sweden, IRB # 2015/143, Regional Ethical Review Board of Uppsala)^36^. Thus, bladder tumors from 939 patients were available for analysis. Demographic, clinical, disease and treatment characteristics are presented in Table 1. The median follow-up for the entire cohort was 5.37 years.

### Immunohistochemical staining of tissue microarrays

Immunostaining was performed using standard protocols. TMAs were deparaffinized in xylene and rehydrated using graded percentages of ethanol followed by antigen retrieval with citric acid buffer (pH 6.0, 95 °C for 20 min). The slides were treated with 3% hydrogen peroxide in water to block endogenous peroxidase activity. Staining for PAI-1 was conducted using rabbit anti-PAI-1 antibody (HPA050039; 1:100 dilution in blocking buffer, Sigma-Aldrich). Antibodies specificity information can be found in manufactures’ datasheet or our previous publication^37^. Biotin-labeled horse anti-mouse IgG (2 µg/ml in blocking buffer, Vector Laboratories) was used as the secondary antibody. Immunoreactive signals were amplified by formation of avidin-biotin peroxidase complexes and visualized using 3, 3ʹ- diaminobenzidine (DAB). Nuclear counterstaining was conducted with hematoxylin.

The expression level of PAI-1 was scored by assigning a proportion score and an intensity score^18,38–40^. The estimated proportion (0 = 0% of cells; 1 = 1% to 40%; 2 = 41% to 75% and 3 = 76% to 100%) and the average intensity (0 = none; 1 = weak; 2 = intermediate and 3 = strong) of immunoreactive tumor cells were assessed. The proportion and intensity scores were combined to obtain a total staining score for each protein, which ranged from 0 to 6. Thus, the protein expression levels were determined based on the total PAI-1 staining score as follows: none = 0, low = 1 or 2, moderate = 3 or 4, high = 5 or 6. Two investigators (OTMC, YS), who were blinded to the clinicopathologic data and clinical outcomes, scored each core. When there was a discrepancy in the scoring, a third investigator reviewed consensus scoring was obtained.

Human lung was used as a positive control and omitting the primary antibody served as the negative control.

### TCGA

Bladder urothelial carcinoma Illumina Hi-Seq counts from The Cancer Genome Atlas (TCGA) were downloaded from the Genomic Data Commons (GDC) data portal, and corresponding clinical annotation including survival information was accessed via the TCGA Clinical Data Resource. The data was comprised of 430 samples from TCGA with gene transcriptome data of which 404 patients had valid survival data (19 normal and 411 cancer)^41^. These data are an open resource, and no ethical issues were involved.

### Survival analysis

Kaplan–Meier curves were used to distinguish the connection between the individual biomarkers (low vs. high expression) within the signature(s) and prognosis. High expression was defined as ≥ median, while low expression was < median. Then, Kaplan–Meier curves were used to distinguish the connection between the entire signature (i.e., composite of biomarkers, low vs. high expression) within the signature(s) and prognosis.

### Cell lines

Human bladder cancer cell lines; RT112 and UM-UC-3 cell lines were provided by the Pathology Core of the Bladder Cancer SPORE at MD Anderson Cancer Center. Cells were authenticated by DNA fingerprinting using the AmpFISTR Amplification or AmpFISTR Profiler PCR Amplification protocols (Life Technologies). Cell lines were maintained in RPMI 1640 or DMEM media as previously described^42^.

### Immunofluorescence (IF) microscopy

RT112 and UM-UC-3 cells were seeded on chamber slides (#154461; Nalgene Nunc International, Rochester, NY) at 50% confluency. The following day, cells were washed with PBS and fixed in 4% paraformaldehyde at room temperature for 15 min. Cells were permeabilized and blocked with 1% BSA and 0.5% Triton X-100 in PBS at room temperature for 30 min. Chamber slides were incubated with anti-PAI-1 antibody (H-135; Santa Cruz Biotechnology, Santa Cruz, CA, 1:50 dilution) diluted in 1% BSA and 0.5% Triton X-100 in PBS at room temperature for 1 h. Cells were washed with PBS and subsequently treated with anti-rabbit DyLight 633 antibody (Catalog #: 35562; Thermo Fisher Scientific, Waltham, MA, 1:280 dilution) at room temperature for 1 h in the dark. Chamber slides were washed and mounted with mounting media containing DAPI (#17984-24; Electron Microscopy Sciences, Hatfield, PA). IF images were captured using a Leica TCS SP5 confocal fluorescence microscope at 400X magnification (Leica Microsystems, Bannockburn, IL).

### Cellular fractionation

Cell fractionation was performed as previously described^43^. Briefly, plated cells were trypsinized and collected by centrifugation at 400 × *g* for 5 min, then washed with ice-cold DPBS. The cells were solubilized in ice-cold 0.1% Nonidet P (NP) 40-PBS containing protease/phosphatase inhibitor. Cytosolic and nuclear fractions were separated by centrifugation at 10,000 × *g* for 3 min at 4 °C. The nuclear pellet was washed once in 0.1% NP40-PBS and centrifuged at 10,000 × *g* for 3 min at 4 °C. The nuclear pellet was resuspended either in RIPA buffer for western blotting assays, or in ChIP buffer within MAGnify™ Chromatin Immunoprecipitation System (Cat No. 492024, Thermo Fisher Scientific, Waltham, MA) for use in immunoprecipitation and ChIP studies. Thirty micrograms of protein from all fractions was analyzed by immunoblot analysis.

### Immunoblotting

Cell lysis and immunoblotting were performed using standard protocols as previously described^17,18,38^. Antibody details are available in Supplementary Material.

### ChIP-sequencing (ChIP-seq)

#### ChIP and ChIP-PCR

ChIP assay was performed using MAGnify™ Chromatin Immunoprecipitation System (Cat No. 492024, Thermo Fisher Scientific) with anti-PAI-1 antibody (M01, clone 3F2; Abnova, Taipei City, Taiwan) according to manufacturer’s instruction. Each sample was analyzed in triplicate by real-time PCR using specific primers as described in Supplementary Material. Enrichment was determined by using the 2^−ΔCT^ method relative to input DNA. Primer specificity was confirmed by evaluating dissociation curves and independently analyzing amplified product on an agarose gel.

#### ChIP library preparation and RNA sequencing

Library construction was performed using the Illumina TruSeq Nano DNA library preparation kit (Illumina, San Diego, CA, USA). Sample libraries were multiplexed and sequenced on a HiSeq2500 platform (Illumina) using 100 bp paired-reads. An average of 25 million reads were generated from each sample.

#### Computational analysis of ChIP-Seq data

ChIP-Seq data were mapped to the reference human genome (hg19) using Bowtie^44^ (version 1.1.1) with the default parameters. PCR duplicates were removed from all downstream analyses with Picard (http://broadinstitute.github.io/picard/). Enriched regions were identified using MACS2^45^ (version 2.1.0.20140616).

### siRNA transfection

RT112 and UM-UC-3 cells were transfected with a pool of 3 synthesized commercial PAI-1 (sc-36179, Santa Cruz Biotechnology) (RT112^KD-PAI1^ and UM-UC-3^KD-PAI1^) or pool of 3 scrambled negative control siRNA (Scr, sc-37007, Santa Cruz Biotechnology) (RT112^Scr^ and UM-UC-3^Scr^) in 6 well plates with a 100-pmol of siRNA and 9 μl of INTERFERin (Polyplus-transfection Inc., NY, USA) for 72 h according to manufacturer’s instruction.

### RNA-sequencing (RNA-seq) for gene expression analysis

#### Library preparation and sequencing

Total RNA samples were assessed for concentration using a Qubit fluorometer (ThermoFisher Scientific, Waltham, MA) and for quality using the 2100 Bioanalyzer (Agilent Technologies, Santa Clara, CA). Library construction was performed using the QIASeq Stranded mRNA Select kit (Qiagen, Hilden, Germany). Library concentration was measured with a Qubit fluorometer and library size on a 4200 TapeStation (Agilent Technologies). Libraries were multiplexed and sequenced on a NovaSeq 6000 (Illumina, San Diego, CA) using 75bp single-end sequencing. An average of 30 million reads were generated from each sample.

### RNA-seq data analysis and overlapping with ChIP-seq data

We used cutadapt^46^ to remove low quality reads and trim adapter sequences from FASTQ files. Human reference genome hg38 aligned to the trimmed reads using STAR aligner^47^. The output, BAM files, aimed to measure gene counts using featureCounts^48^. Then, we used Bioconductor packages DESeq2^49^ and EnhancedVolcano^50^ in R programming environment^51^ for statistical analysis and visualization accordingly.

DESeq2 was subsequently used to compare expression of knock-down vs. control samples for both UM-UC-3 and RT112. An information-theoretic gene-set enrichment analysis (TEISER)^52^ was used to examine the distribution of PAI-1 targets among the differentially expressed genes.

### Rapid immunoprecipitation mass spectrometry of endogenous proteins (RIME) assay

#### ChIP

Chromatin was isolated by the addition of lysis buffer, followed by disruption with a Dounce homogenizer. Lysates were sonicated and the DNA sheared to an average length of 300-500 bp. Genomic DNA (Input) was prepared by treating aliquots of chromatin with RNase, proteinase K and heat for de-crosslinking, followed by ethanol precipitation. Pellets were resuspended and the resulting DNA was quantified on a NanoDrop spectrophotometer. Extrapolation to the original chromatin volume allowed quantitation of the total chromatin yield.

An aliquot of chromatin (125 μg) was precleared with protein G agarose beads (Invitrogen). Proteins of interest were immunoprecipitated using 13 μg of antibody against SERPINE1 (Abnova, Cat. # H00005054-M01) and protein G magnetic beads. Protein complexes were washed then trypsin was used to remove the immunoprecipitate from beads and digested the protein sample. Protein digests were separated from the beads and purified using a C18 spin column (Harvard Apparatus). The peptides were vacuum dried using a speedvac.

#### Mass spectrometry

Digested peptides were analyzed by LC-MS/MS on a Thermo Scientific Q Exactive Orbitrap Mass spectrometer linked to Dionex Ultimate 3000 HPLC (Thermo Scientific) and a nanospray FlexTM ion source. The digested peptides were loaded directly onto the separation column Waters BEH C18, 75 micron × 100 mm, 130Å 1.7u particle size. Peptides were eluted using a 100 min gradient with a flow rate of 323 nl/min. An MS survey scan was obtained for the m/z range 340–1600, MS/MS spectra were acquired using a top 15 method, where the top 15 ions in the MS spectra were subjected to HCD (High Energy Collisional Dissociation). An isolation mass window of 1.6 m/z was for the precursor ion selection, and normalized collision energy of 27% was used for fragmentation. A 20 second duration was used for the dynamic exclusion.

#### Database searching

Tandem mass spectra were extracted and analyzed by PEAKS Studio version 8 built 20. Charge state deconvolution and deisotoping were not performed. Database consisted of the Uniprot database (version 180508, 71771 curated entries) and the cRAP database of common laboratory contaminants (www.thegpm.org/crap; 114 entries). Database was searched with a fragment ion mass tolerance of 0.02 Da and a parent ion tolerance of 10 PPM. Post-translational variable modifications consisted of methionine oxidation, asparagine and glutamine deamidation.

#### Criteria for protein identification

Peaks studio built-in decoy sequencing and FDR determination was used to validate MS/MS based peptide and the parsimony rules for protein identifications. A threshold of the − 10 * logp (*p *value) of 20 or greater was applied for the peptide identifications. The weighted sum of 9 parameters for peptide scoring are converted to a p-value which represent the probability of a false identification. Protein identifications were accepted if they could pass the − 10logp of 20 and contained at least 1 identified unique peptide. Proteins that contained similar peptides and could not be differentiated based on MS/MS analysis alone were grouped to satisfy the principles of parsimony. Proteins sharing significant peptide evidence were grouped into protein groups.

#### List filtering

Protein coverage percentage is observed in the raw data file. Final list generation was done by taking all proteins with a spectral count of five and above from each replicate reaction and comparing them in a venn-diagram against IgG control replicates. Proteins unique to both experimental replicates were then applied to the PANTHER database for protein ontology results.

### Statistical analysis

All *in vitro* data are expressed as mean ± standard deviation (SD). Differences between two or more groups were analyzed by a 2-tailed unpaired Student’s *t* test or ANOVA followed by Tukey’s *post-hoc* test. Statistical analyses were performed with GraphPad Prism 7.0 (GraphPad Software, Inc.) and SAS 9.4 (SAS Institute Inc., Cary, NC). A *p* value less than 0.05 was considered significant.

## Supplementary Information

Supplementary Information 1.

Supplementary Information 2.

Supplementary Information 3.

Supplementary Information 4.

Supplementary Information 5.

Supplementary Information 6.

Supplementary Information 7.

Supplementary Information 8.

Supplementary Information 9.

Supplementary Information 10.

Supplementary Information 11.

Supplementary Information 12.

## Data Availability

The datasets generated and/or analysed during the current study are available in the Gene Expression Omnibus (GEO) repository, GSE201515 (https://www.ncbi.nlm.nih.gov/geo/query/acc.cgi?acc=GSE201515) and GSE201517 (https://www.ncbi.nlm.nih.gov/geo/query/acc.cgi?acc=GSE201517).
