# Supplementary material for: PAI-1 is a potential transcriptional silencer that supports bladder cancer cell activity
Source: Sci Rep. 2022 Jul 16;12:12186. doi: 10.1038/s41598-022-16518-3 (PMC9288475; doi:10.1038/s41598-022-16518-3)
Supplement: Supplementary file 1 — Supplementary Information 1. [file 41598_2022_16518_MOESM1_ESM.docx]

**Supplementary Materials and methods**

**Immunoblotting Antibodies**

For immunoblots, proteins were transferred to polyvinyl difluoride membranes (Bio-Rad) and stained using a mouse anti-human PAI-1 antibody (BD Biosciences, Cat# 612025, mouse monoclonal, dilution 1:1000), rabbit anti-Lamin A antibody (Santa Cruz Biotechnology, Cat# sc-20680, dilution 1:500), and goat anti-human β-actin antibody (Santa Cruz Biotechnology, Cat# sc-1615, dilution 1:2000). The immunoblots were visualized using Clarity Western enhanced chemiluminescence substrate (Bio-Rad, Hercules, CA) and a digital darkroom (C-Digit Blot Scanner, Li-Cor Biosciences, Lincoln, NE).

**List of Primers:**

| Genes | Forward Primer | Reverse Primer | Size product (bp) |
| --- | --- | --- | --- |
| **APC** | GTCCCTCCAGCCTCTCTGTA | GCTGTGAGTTCAGCACTCCT | 102 |
| **CIRBP** | CTGGTCACGCCCCCTC | CGACTATTGGCCGAGACGTT | 64 |
| **DOCK2** | CAAAGCTCATGCTGAGGGGA | GAGTTTCCTGATTGCCGTGC | 66 |
| **HDAC2** | GCAGAGTGGAACAGCTAGGG | AGGGGGCCTATTACGGATGT | 300 |
| **HMGB1** | GACATGCTGTGAAACTCGATCA | AGGGCGACACTATGGGATCA | 297 |
| **LIN9** | AGGAACAGTGACACTACAAGGT | CCTTGGGCTTCTTTCAATTTCGT | 93 |
| **LPXN** | GACACCCCGAGAAAGGTACG | ATTGCTGGTCTGATGGGAGG | 79 |
| **LTBR** | CTTCCCTCGGCTGGGC | CCTCCCTTCTTCGGAAGCC | 66 |
| **NFKB1** | AAAGACACATCCGGACCTCG | TGTAAGAGTTCCCCTCCGGT | 118 |
| **PLK2** | TTGACAACCGTGGGCCTTTG | TGGAAACTCACAATCGGCATC | 63 |
| **PTGS2** | GCCAAGCACTTTTGGTGGAG | GGGACAGCCCTTCACGTTAT | 91 |
| **SPP1** | AGCACAGTGTGAAGTTCGGT | GCATATAGTCACCCGGACCC | 293 |

**ChIP – quantitative PCR**

**Supplementary Figure Legends**

**Supplementary Figure S3. Original western blot images for Figure 2B.**

(A) Subcellular localization of PAI-1 protein in UM-UC-3 cell line, (B) Subcellular localization of Lamin A protein (nuclear marker) in UM-UC-3 cell line, (C) Subcellular localization of β-actin protein (cytoplasm marker) in UM-UC-3 cell line, (D) Subcellular localization of PAI-1 protein in RT112 cell line, (E) Subcellular localization of Lamin A protein in RT112 cell line, and (F) Subcellular localization of β-actin protein in RT112 cell line. Note – Images for RT112 Lamin A and β-actin demonstrate partial protein markers because of stripping and re-probing the membrane resulting faded protein markers. In addition, in our detection protocol (Clarity™ Western ECL Substrate from Bio-Rad followed by the scan using C-Digit from LI-COR), the edges of membrane were not visible in most images.
