# Supplementary material for: PAI-1 is a potential transcriptional silencer that supports bladder cancer cell activity
Source: Sci Rep. 2022 Jul 16;12:12186. doi: 10.1038/s41598-022-16518-3 (PMC9288475; doi:10.1038/s41598-022-16518-3)
Supplement: Supplementary file 5 — Supplementary Information 5. [file 41598_2022_16518_MOESM5_ESM.docx]

| **Supplementary Table S4 – List of proteins identified by RIME** | |
| --- | --- |
| **Symbols** | **Protein name** |
| RBM27 | RNA-binding protein 27 |
| ZC3H14 | Zinc finger CCCH domain-containing protein 14 |
| RBM14 | RNA-binding protein 14 |
| PABPN1 | Polyadenylate-binding protein 2 |
| RBM26 | RNA-binding protein 26 |
| SRRT | Serrate RNA effector molecule homolog |
| ZFC3H1 | Zinc finger C3H1 domain-containing protein |
| SRSF7 | Serine/arginine-rich-splicing factor 7 |
| ILF2 | Interleukin enhancer-binding factor 2 |
| NUDT21 | Cleavage and polyadenylation specificity factor subunit 5 |
| RNPS1 | RNA-binding protein with serine-rich domain 1 |
| SERPINE1 | Plasminogen activator inhibitor 1 |
| SRSF6 | Serine/arginine-rich splicing factor 6 |
