# Supplementary material for: PAI-1 is a potential transcriptional silencer that supports bladder cancer cell activity
Source: Sci Rep. 2022 Jul 16;12:12186. doi: 10.1038/s41598-022-16518-3 (PMC9288475; doi:10.1038/s41598-022-16518-3)
Supplement: Supplementary file 6 — Supplementary Information 6. [file 41598_2022_16518_MOESM6_ESM.pdf]

A

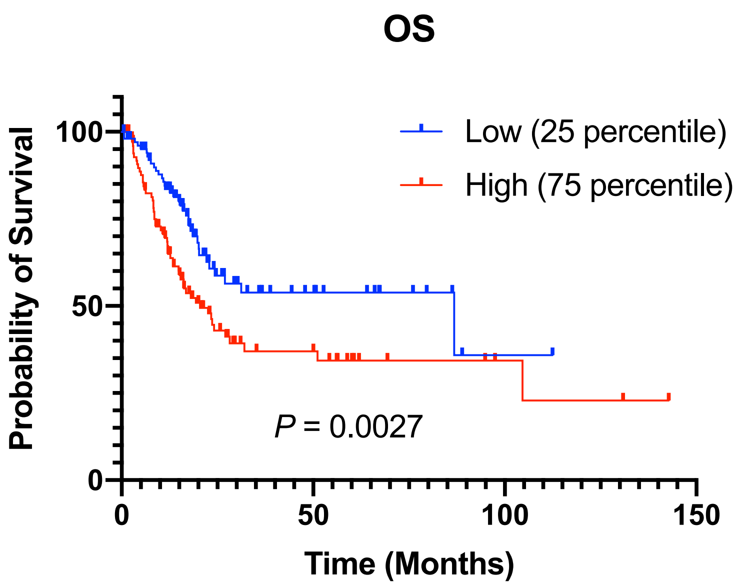

B

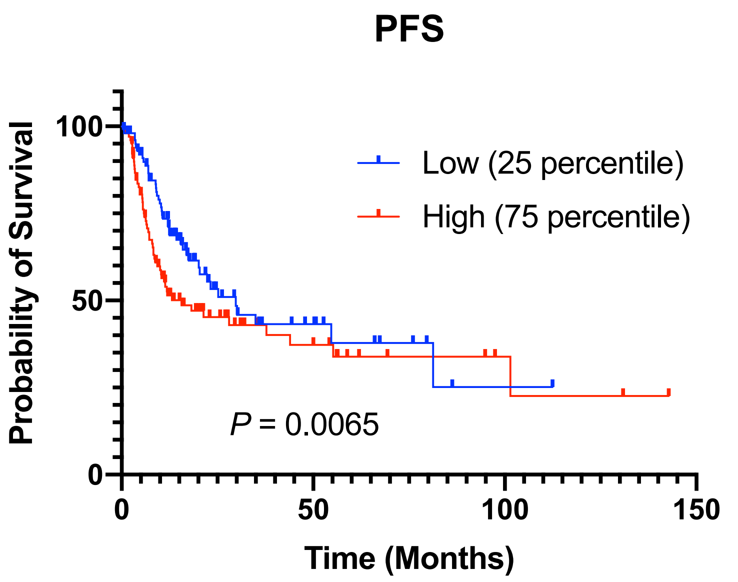

Supplementary Figure S1. Correlation between PAI-1 expression and overall survival (A) and progression-free survival (B) in TCGA BLCA (n = 412).

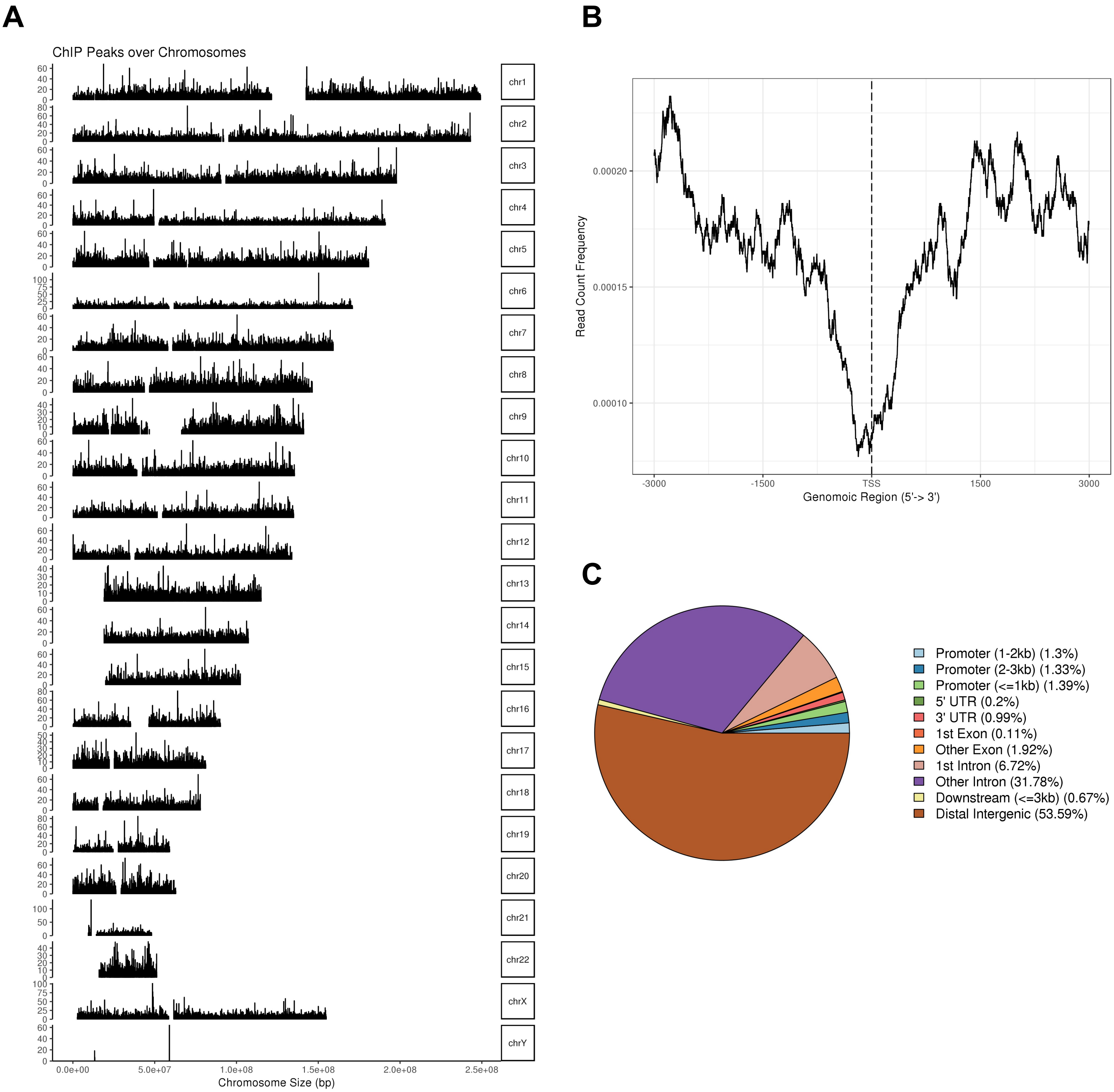

Supplementary Figure S2. ChIP-seq profiling in RT112 cells.
(A) Histogram demonstrating distribution of PAI-1 binding across the genome in RT112 cells. The frequency of PAI-1 binding across chromosomes was calculated by dividing the number of probe sets per chromosome by the number of probe sets bound by PAI-1 with FDR <0.2. (B) Histogram demonstrating relative PAI-1 bound peak location with respect to chromosome region in RT112 cells. (C) Pie chart of the genomic location distribution of PAI-1 in RT112 cells. This plot shows the percentage for each genomic location category. The categories are sorted by descending percentage.
