# Supplementary figures and images for: PAI-1 is a potential transcriptional silencer that supports bladder cancer cell activity
Source: Sci Rep. 2022 Jul 16;12:12186. doi: 10.1038/s41598-022-16518-3 (PMC9288475; doi:10.1038/s41598-022-16518-3)

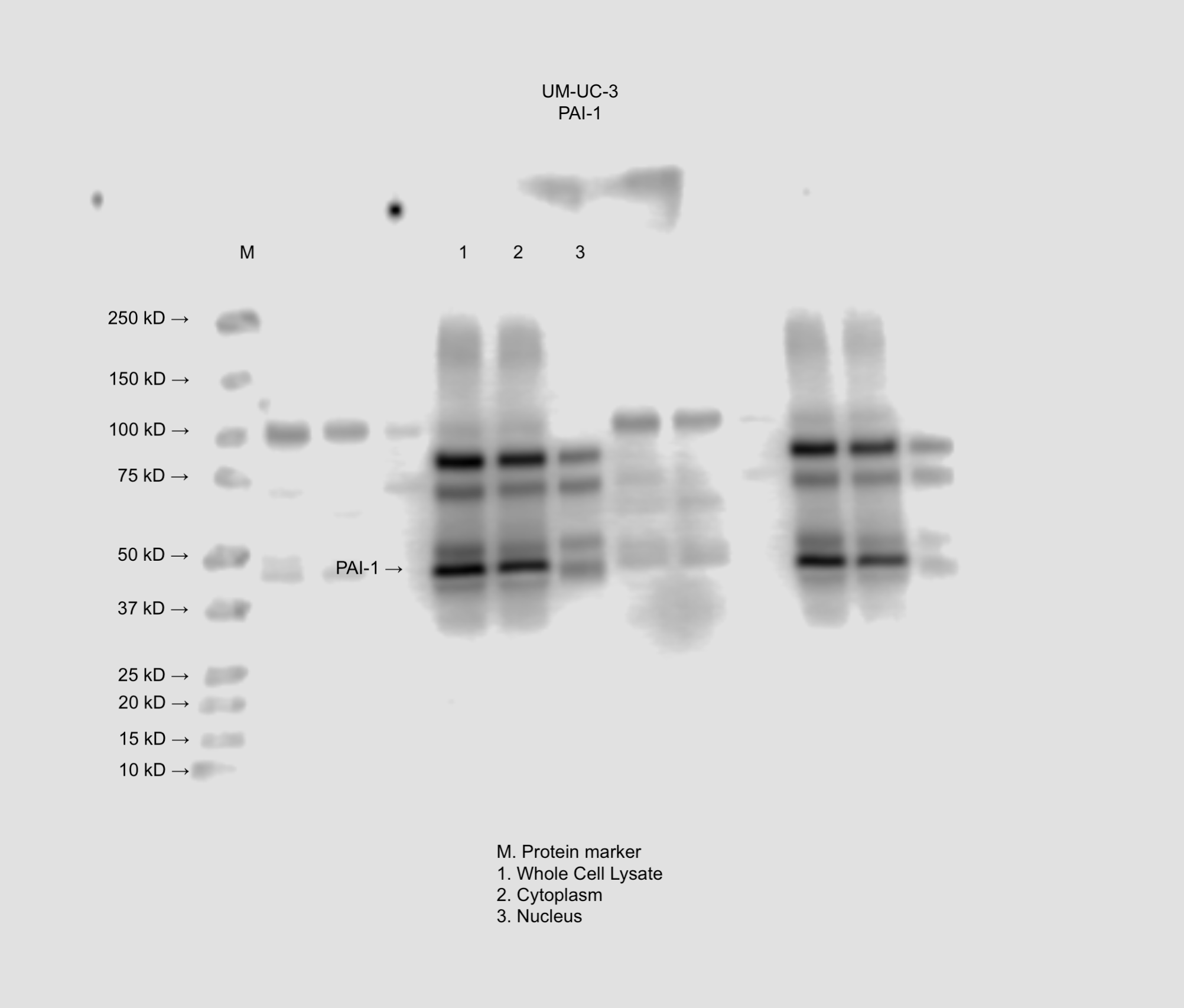

Supplement: Supplementary file 7 — Supplementary Information 7. [file 41598_2022_16518_MOESM7_ESM.tif]

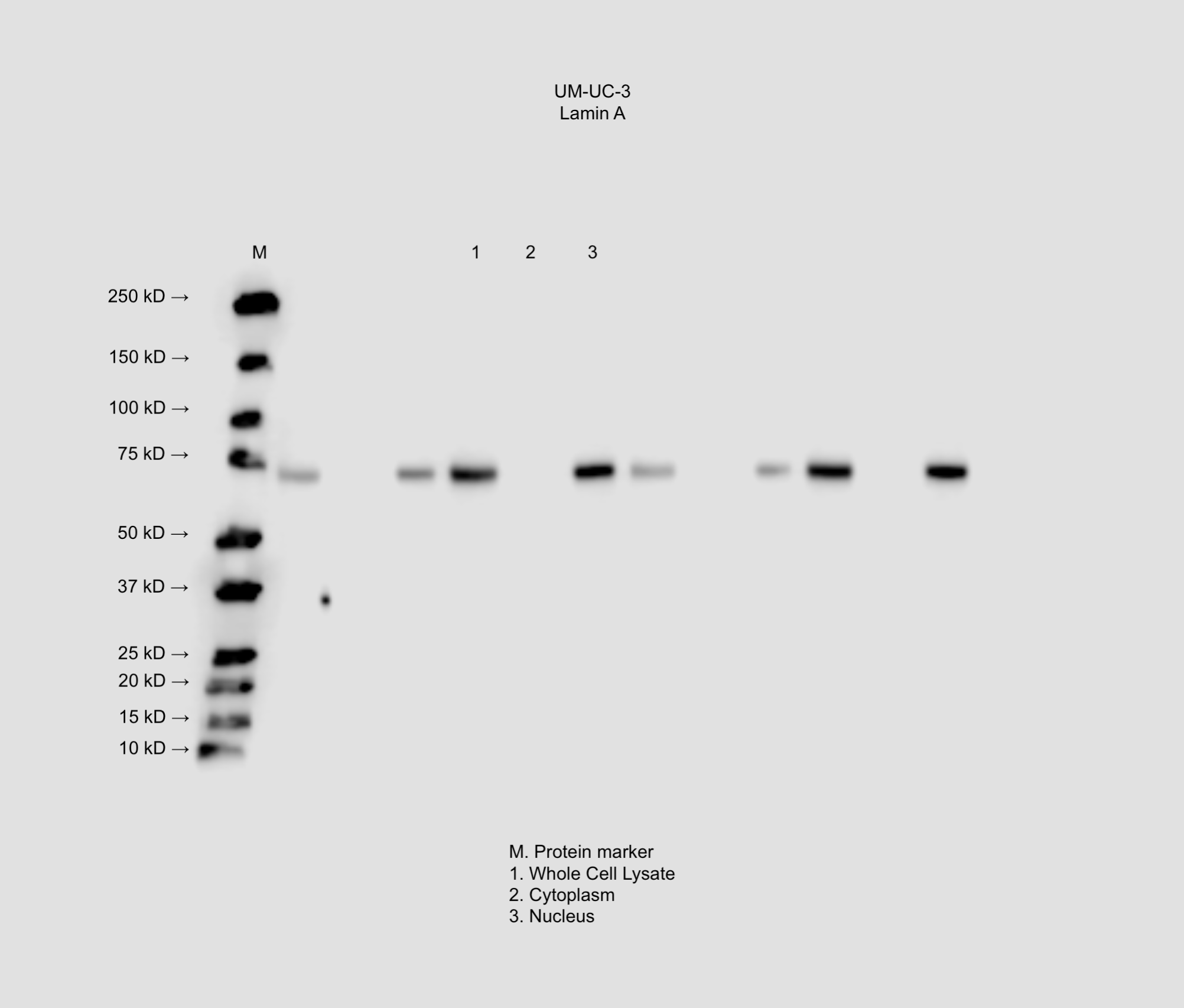

Supplement: Supplementary file 8 — Supplementary Information 8. [file 41598_2022_16518_MOESM8_ESM.tif]

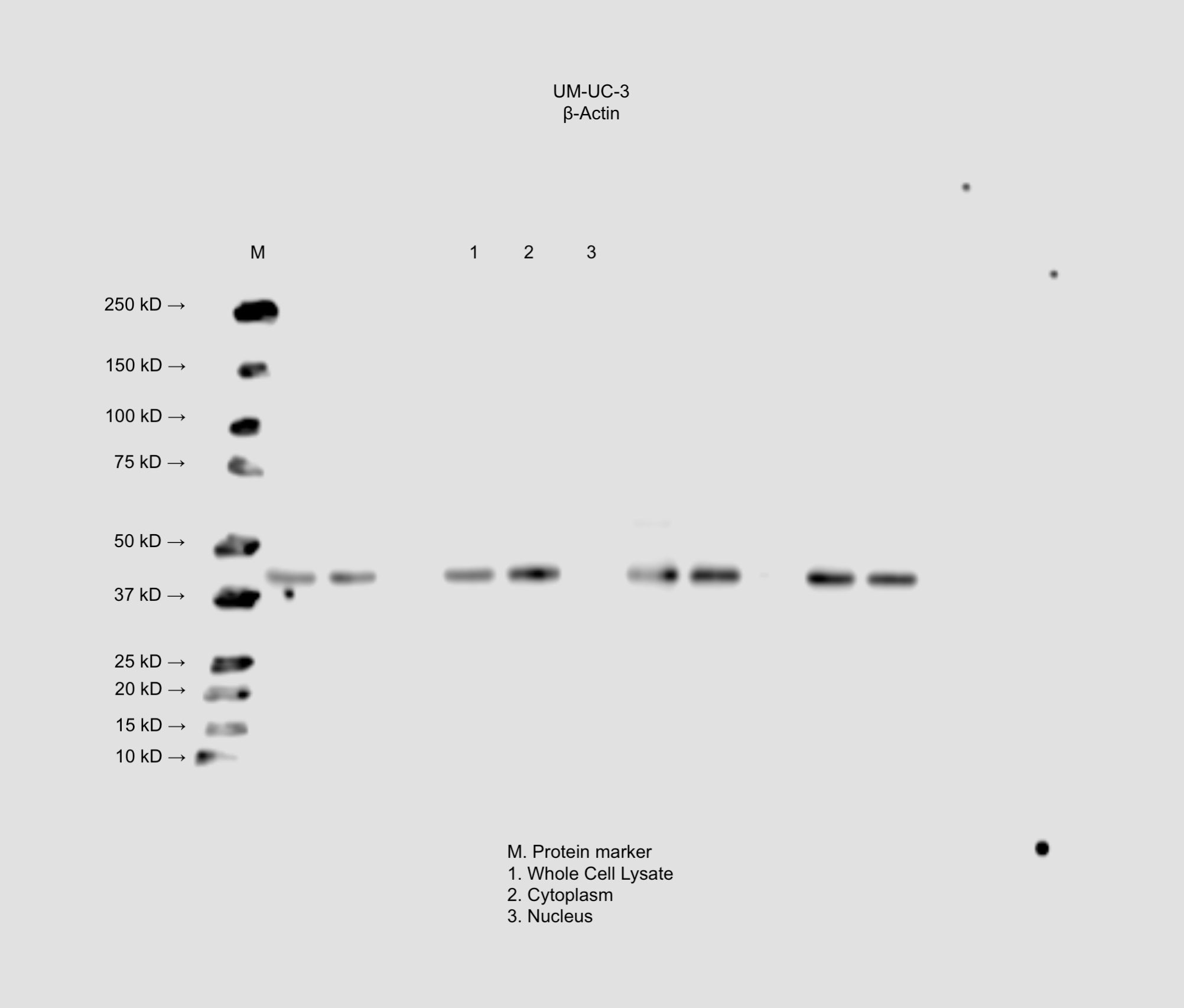

Supplement: Supplementary file 9 — Supplementary Information 9. [file 41598_2022_16518_MOESM9_ESM.tif]

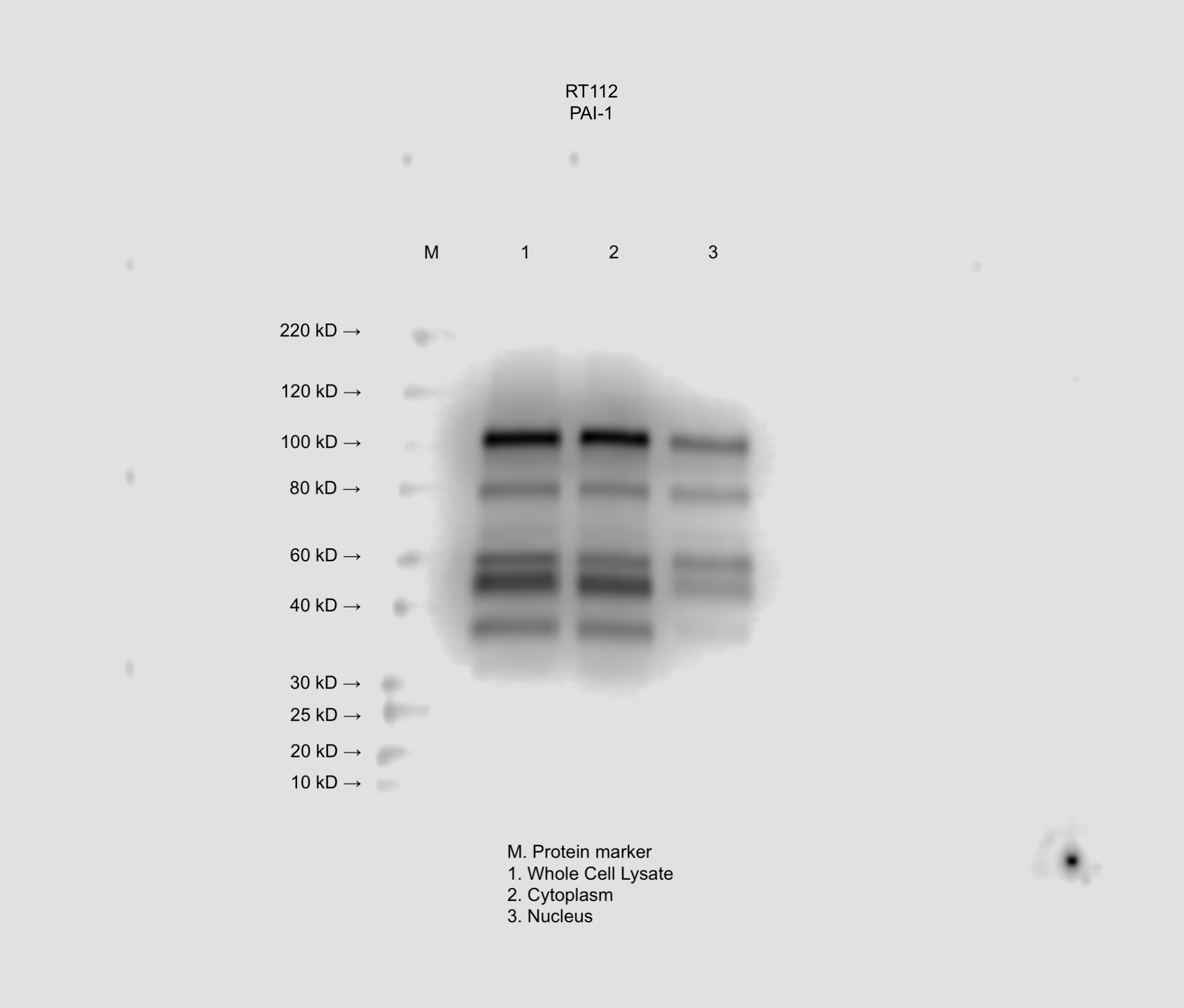

Supplement: Supplementary file 10 — Supplementary Information 10. [file 41598_2022_16518_MOESM10_ESM.tif]

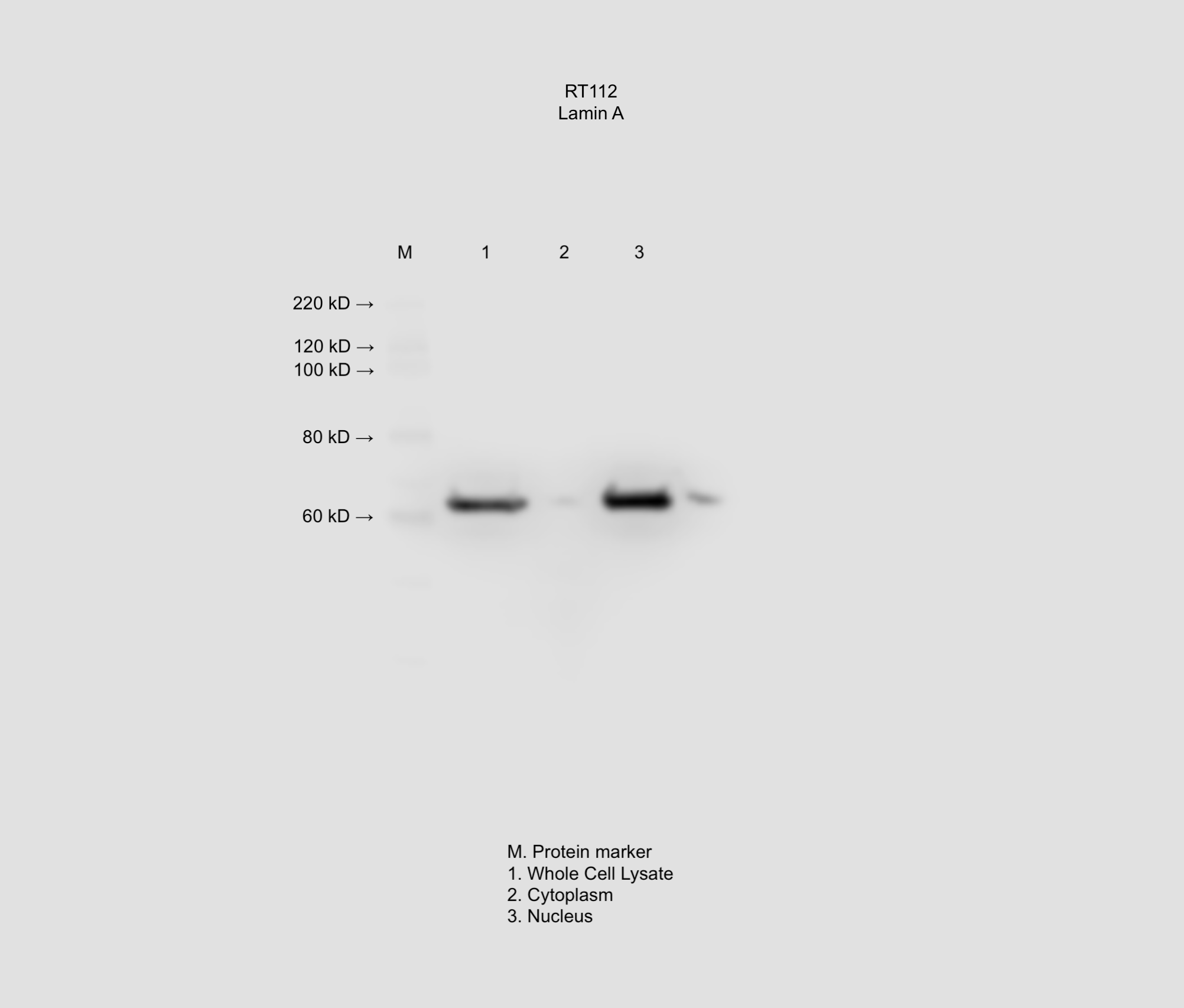

Supplement: Supplementary file 11 — Supplementary Information 11. [file 41598_2022_16518_MOESM11_ESM.tif]

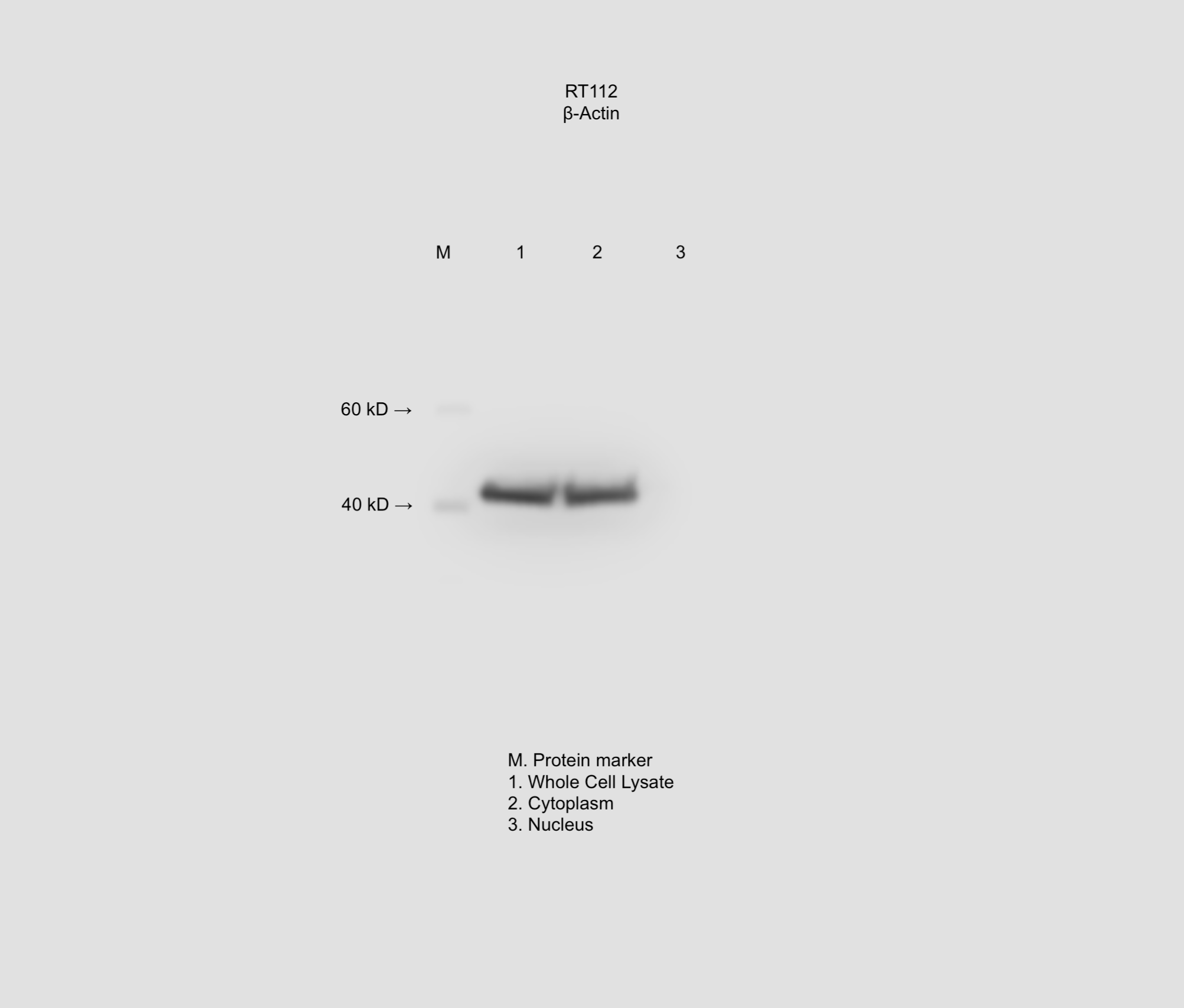

Supplement: Supplementary file 12 — Supplementary Information 12. [file 41598_2022_16518_MOESM12_ESM.tif]
